# Systematic review of transarterial chemoembolization for desmoid tumors: a promising locoregional treatment for challenging tumor

**DOI:** 10.1007/s12672-025-03007-y

**Published:** 2025-07-01

**Authors:** Abdulrahman Alaseem, Naif Alsaikhan, Abdulaziz M. AlSudairi, Yazeed A. Alsehibani, Mohammed N. Alhuqbani, Zyad A. Aldosari, Omar A. Aldosari, Abdulaziz Almuhanna, Ibrahim S. Alshaygy

**Affiliations:** 1 Department of Orthopedic Surgery, College of Medicine, King Saud University, Riyadh, Saudi Arabia; 2 Department of Radiology, College of Medicine, King Saud University, Riyadh, Saudi Arabia; 3 Orthopedic Surgery Department, King Saud Medical City, Riyadh, Saudi Arabia

**Keywords:** Desmoid, Chemoembolization, Oncology, Soft tissue tumors, Interventional radiology

## Abstract

**Background:**

Desmoid tumors are rare, locally invasive nonmetastatic tumors that arise from soft tissues. Due to the complexity of desmoid tumor therapy, each patient’s particular needs and preferences must be considered in a multidisciplinary strategy. To provide innovative therapeutic approaches and improve the results for patients with desmoid tumors, ongoing research, and collaboration within the medical community are essential.

**Search methods:**

A comprehensive search of three databases was conducted from inception to June 2024 to identify all relevant cases of chemoembolization for desmoid tumors. Data from these cases were systematically extracted and analyzed to assess the procedure’s overall efficacy and safety concerns.

**Search results:**

A total of 6 studies were identified in the literature with a combined sample size of 43 patients. Doxorubicin was the predominant chemotherapeutic agent administered using drug-eluting microbeads (DEB-TACE). The magnitude of size reduction varied from 21 to 97%. Most studies observed a decrease in size exceeding 50%. All 43 patients observed a decrease in symptoms following the procedures.

**Conclusions:**

This review emphasizes that chemoembolization shows potential as a reliable and low-risk approach for treating desmoid tumors. Chemoembolization can be used especially when the tumors are large or located in anatomically complex areas with clearly defined blood vessels.

## Introduction

### Background and epidemiology

Desmoid tumors, also known as aggressive fibromatosis or deep fibromatosis, are rare, locally invasive nonmetastatic tumors that arise from soft tissues [1, 2]. It has the potential to proliferate multifocally from mesenchymal stem cell progenitors [3]. Desmoid tumors differ from other types of tumors in that they have unpredictable growth patterns, an infiltrative nature, and a propensity to come back after surgical removal. With an estimated prevalence of two to six per million population, desmoid tumors are more common in young adults, particularly females between the ages of 30 and 40 who are of reproductive age [2, 4, 5]. These tumors can occur anywhere in the body, with a predilection to the abdominal wall, mesentery, shoulder girdle, and extremities [1, 5, 6].

### Current management strategies

**Fig. 1:**
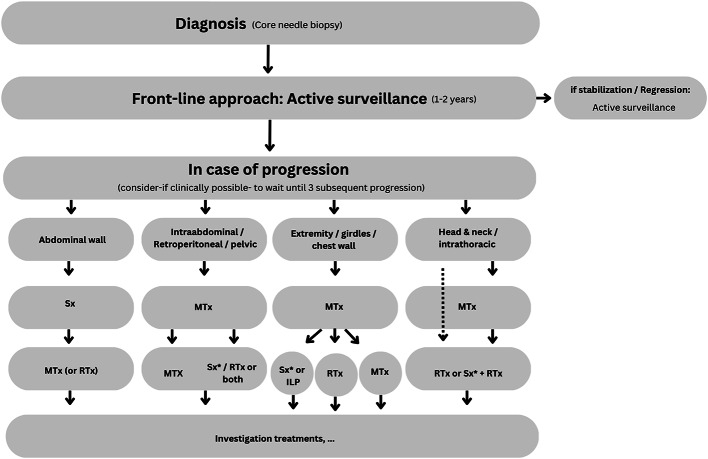
The Desmoid Tumor Working Group proposed treatment algorithm. *Sx* surgery, *MTx* medical treatment, *RTx* radiotherapy, *ILP* isolated limb perfusion

The primary objectives in the overall management strategy of these tumors are pain control and improving patient’s quality of life [7]. A consensual treatment plan for desmoid tumors has been sought by the Desmoid Tumor Working Group in Europe to solve this conundrum, as shown in Fig. 1 [5, 8]. This method comprises an algorithm with a stepwise framework depending on patient preferences, disease progression and symptoms. The current treatment options for desmoid tumors encompass active surveillance, surgery, radiation therapy, systemic therapy and local therapies such as cryoablation and radiofrequency ablation [9–11]. However, each modality has its own limitations and potential side effects, making the selection of an appropriate treatment strategy complex and personalized for each patient.

Active surveillance has become the standard of care for the majority of patients, taking into account the fact that 50–60% of desmoid tumors do not exhibit growth after diagnosis, and a significant proportion may even shrink following the initial presentation [7]. Previously, surgical excision was thought to be the best course of action for desmoid tumors, but it has been linked to a high rate of recurrence, functional impairment, and a risk of significant morbidity and complications [4]. Surgery may be considered in cases where morbidity is minimal, and patients are symptomatic. In certain cases, radiation treatment may be successful, but it is limited due to the possibility of long-term harm, particularly in younger people [4]. Systemic therapy, such as tyrosine kinase inhibitors, hormonal therapies, anthracycline-based regimens, and nonsteroidal anti-inflammatory medications (NSAIDs), have shown varying response rates and might be appropriate for certain individuals [1]. These systemic therapies are typically utilized for tumors located outside the abdominal wall, such as those in the head and neck region [5]. Despite being minimally invasive, local treatments may not be as effective in treating larger or deeply rooted tumors.

Due to the complexity of desmoid tumor therapy, each patient’s particular needs and preferences must be taken into account in a multidisciplinary, tailored strategy. To provide innovative therapeutic approaches and improve the results for patients with desmoid tumors, ongoing research, and collaboration within the medical community are essential.

In light of these limitations and the need for alternative treatment options, the purpose of this review is to introduce chemoembolization as a prospective alternative for the management of desmoid tumors. Chemoembolization is not currently considered a standard of care for the treatment of desmoid tumors. While promising, the available evidence remains limited, and there is a pressing need for high-quality, international, prospective, multicenter studies with larger patient cohorts to evaluate the safety, efficacy, and long-term outcomes of this method in desmoid tumor management. Chemoembolization combines the targeted delivery of chemotherapeutic agents with the embolization of tumor-feeding blood vessels, maximizing local drug concentrations and minimizing systemic toxicity. This review seeks to cast light on the role of chemoembolization in the management of desmoid tumors by emphasizing the available evidence and discussing its potential benefits and drawbacks. Through this review, we aim to shed light on the current state of knowledge concerning chemoembolization in desmoid tumors and to contribute to the ongoing discussion on optimizing therapeutic strategies for this difficult disease.

### Technique and procedure

The concept behind chemoembolization is leveraging the DFs tissue’s innate hypervascularity as means for endovascular catheter navigation to deliver doxorubicin locally [19]. To enable microbead-loaded doxorubicin eluting embolization (DEE), doxorubicin includes a protonated amine group that can be ionically linked to sulfonate-coated hydrogel microbeads [25].

**Fig. 2:**
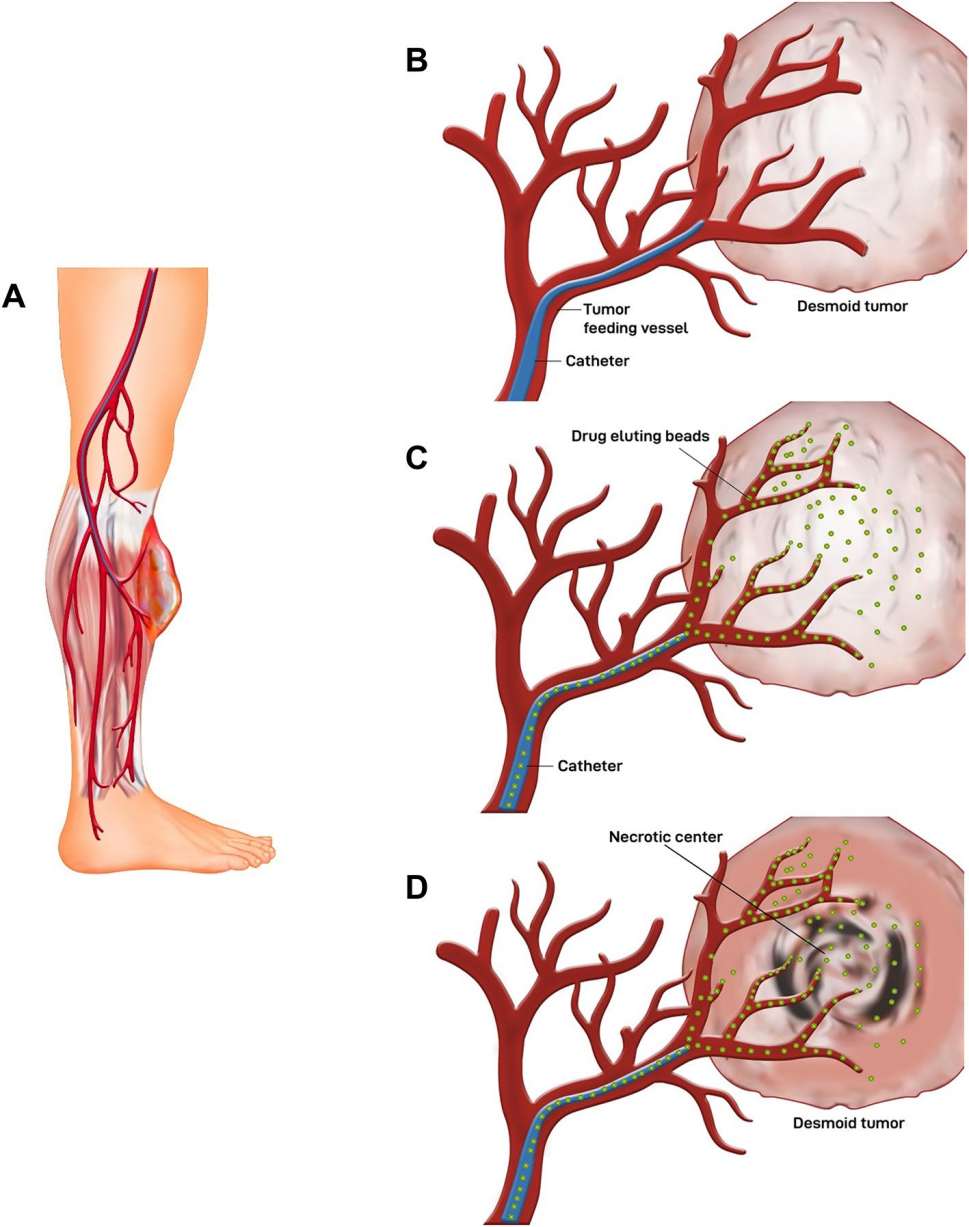
**A** Image-guided access to the tumor through a catheter using major vessels for access, **B** Superselective catheterization of the arterial branch feeding the tumor, **C** Occlusion of feeding artery by drug-eluting microsphere-loaded with a chemotherapeutic agent, **D** chemotherapeutic agents alongside tissue ischemia leads to necrosis in the targeted tissue

Technique and procedure [19]:

1. Professional and anesthesia: The procedure is performed by an interventional radiologist under conscious sedation to ensure the safety of the patient throughout the procedure.
2. Accessing the arterial system: Depending on the location of the tumor, a suitable arterial access is chosen. Femoral artery access is preferred for most embolization procedures in general. Selective catheterization of the artery of interest is typically done using 4 Fr or 5 Fr angiographic catheters. Super-selective catheterization of the arteries directly feeding the tumor is done using 2.7 Fr–1.6 Fr coaxial micro-catheters.
3. Selecting the tumor-feeding artery: Once selected, digital subtraction angiography (DSA) is performed. Desmoid tumors would show a typical contrast blush characteristic of hyper-vascular tumors. Intra-procedural cone beam CT (CBCT) angiography can be used to facilitate navigation of the arterial tree and to provide a perfusion map of the selected artery prior to treatment.
4. Administering chemo-embolizing agents: Administering a potent chemotherapy treatment, such as doxorubicin, directly to the desmoid tumor via the catheter. Concurrently, the catheter is used to introduce embolic agents, which are small particles or beads containing chemotherapy, into the blood arteries that provide nourishment to the tumor. The embolic beads and chemotherapy are concurrently introduced into the bloodstream, obstructing the tumor’s blood supply and providing a prolonged and targeted therapeutic effect. This strategy minimizes the risk of harming healthy tissues, decreases the occurrence of side effects throughout the body, and maximizes the overall efficiency of the treatment by cutting off the tumor’s supply of necessary oxygen and nutrients while focusing chemotherapy specifically at the tumor location. The DEB-TACE approach utilizes standard angiographic procedures to assure accurate and focused distribution, hence improving the outcome of the surgery Fig. 2.
5. Radiological endpoints of embolization: In DEB-TACE, achieving angiographic stasis or near-stasis stands out as a critical milestone, representing the cessation or significant reduction of blood flow within the targeted vessels supplying the desmoid tumor. This radiological endpoint is indicative of the successful embolization of the tumor’s vascular supply, a pivotal objective in chemoembolization procedures.
6. Post-procedure imaging: Once the chemoembolization is complete, the catheter is removed, and the incision is closed. Post-procedure imaging, such as a CT scan or MRI, may be performed to assess the treatment’s success and detect any complications.
7. Objectives and Assessments: radiological response is usually the primary endpoint. MRI should be obtained at baseline and then at intervals. A heterogeneously hyperintense T2 or a short TI inversion recovery (STIR) signal within interspersed hypo-intense bands are defining MRI characteristics of DFs [19]. Histologically, the amount of cellularity versus fibrotic matrix in DFs is correlated with the ratio of a T2 signal to hypo-intense bands [20, 25, 26]. Reliable indicators of DFs responses to systemic treatment include decreased tumor volume and reduction of T2 intensity [27, 28].

## Materials and methods

### Literature search strategy

**Fig. 3:**
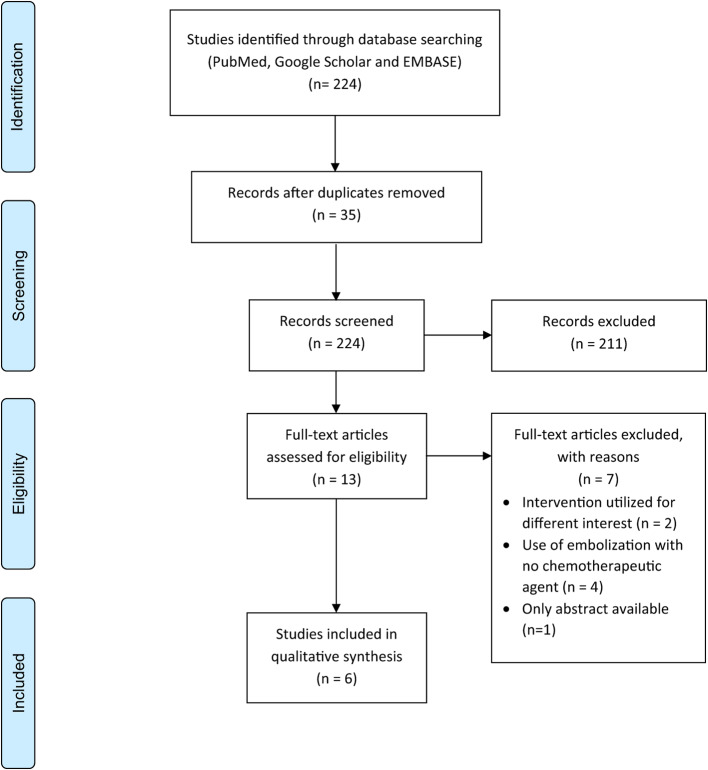
PRISMA flowchart of articles selection through different screening and eligibility phases for inclusion in systematic review

This systematic review has followed the strict requirements outlined by the Preferred Reporting Items for Systematic Reviews and Meta-Analyses (PRISMA), guaranteeing a thorough and transparent procedure for combining evidence. The main aim of this study was to thoroughly assess and report the existing literature on the effectiveness of transarterial chemoembolization in treating desmoid tumors. The exhaustive literature search covered a wide range of electronic databases from their beginning to December 2023. The databases examined for relevant research encompassed Medline, Google Scholar, and EMBASE. The inquiry employed a methodical approach, using a dual-term strategy that included different synonyms to maximize inclusivity and comprehensiveness. The selected search phrases focused on two main concepts: desmoid tumor or aggressive fibromatosis (across all fields) and transarterial chemoembolization or TACE (across all fields). The conscious decision was made to encompass a broad range of research that specifically examines the use of transarterial chemoembolization for desmoid tumors. The detailed search approach and string are thoroughly documented in Appendix A. This systematic review intentionally chose to thoroughly analyze both past and present research without any time limitations, as the scarcity of available information was expected. The flow of study selection is shown in Fig. 3.

### Eligibility criteria

The eligibility criteria for our study comprised research publications that satisfied the following criteria: [1] The clinical and therapeutic features of chemoembolization in desmoid tumors will be considered [2]. There are no constraints on the timing for publication [3]. Publications in any language will be included. If deemed appropriate, the utilization of translation services offered by competent translators will be employed to guarantee a precise understanding of the text [4]. The presence of full-text articles that can be accessed, and [5] the inclusion of reports on randomized clinical trials, observational studies, case-control studies, case series, or case reports [6]. Documentation of clinical effectiveness, radiographic improvement, and adverse reactions. Our investigation employed various exclusion criteria. Firstly, studies lack relevant information on chemoembolization for desmoid tumors. Furthermore, papers without sufficient data or containing critical methodological faults were excluded. In addition, studies that have not been published, conference abstracts, systematic reviews, meta-analyses, animal experiments, narrative reviews, and editorial pieces were not taken into account.

### Search screening

The identified records were transferred to reference management software, specifically Endnote X9, for subsequent analysis and identification of any duplicates. The abstracts of the listed publications were initially evaluated using the Rayyan search engine to facilitate abstract screening. The approach adhered to the predetermined inclusion and exclusion criteria and engaged two autonomous reviewers. To address any discrepancies among the reviewers, a third assessor conducted an unbiased study of the selected articles. Subsequently, a comprehensive examination of the full texts of the studies was conducted to ensure adherence to the predetermined criteria for inclusion and exclusion, prior to commencing the data extraction process. Two reviewers independently obtained and evaluated the full texts of the papers that met the criteria for probable eligibility, in order to determine their inclusion. Supplementary publications were chosen from the bibliographies of the incorporated studies. Disagreements were resolved by reaching a consensus or, if necessary, including a third reviewer.

### Quality and Bias risk assessment

The methodological quality of the research included will be thoroughly assessed using established tools, that is the Cochrane Risk of Bias tool for randomized controlled trials, and the JBI Critical Appraisal Checklist for case series and reports identified. An extensive evaluation will encompass the examination of selection, performance, detection, attrition, reporting, and other biases. The review process will be carried out autonomously by two reviewers, and any inconsistencies will be resolved by conversation. The specifics of quality evaluation will be presented in Appendix B.

### Data extraction

The collected data from the selected articles included a wide range of essential parameters for a detailed study of the research. The foundational aspects of each study, such as the study title, authorship details, publication year, place of origin, and study design, was initially extracted. Demographic insights collected included the number of patients, gender distribution, age profiles, follow-up duration, and the classification of desmoid tumors as either sporadic or hereditary. The parameters of the desmoid tumor, including its size, volume, and location, were methodically documented, along with the indications for chemoembolization. The procedural-related data encompassed the total number of sessions, the number of agents employed, the dosages administered, and the duration of hospitalization. The adverse effects associated with chemoembolization were carefully examined and reported, and the responses in terms of oncological and clinical outcomes were assessed by considering the reduction in size and necrosis percentage. This analysis resulted in a thorough compilation of various topics included in the analyzed research.

### Statistical analysis

No statistical analysis was done. The absence of a meta-analysis can be attributed to the scarcity of the data and the overall variability observed in the portrayal and presentation of the data.

## Results

Transarterial chemoembolization has recently come to be recognized as a viable therapeutic option for the treatment of desmoid tumors (Table 1). Furthermore, to extend the duration of the treatment period of chemotherapeutic agents within tumor beds, transarterial chemoembolization may also be used alongside embolization. This has the additional advantage of lowering the level of chemotherapy that is present in the systemic circulation at any given point in time [13].

In a case report involving a 19-year-old male patient who experienced a recurrence of a desmoid tumor in the shoulder after undergoing surgery [15]. The initial treatment approach involved arterial embolization, but nevertheless, no improvement in symptoms was observed. In the context of second-line therapy, a combination of 20 mg of epirubicin, 50 mg of cisplatin, and 250 mg of 5-FU was administered via infusion into tumor-related arteries. Additionally, embolization was carried out using a super-absorbent polymer microsphere. Following a single treatment session, a decrease in tumor size and a relief of symptoms was observed. The identical therapeutic intervention was administered on three separate occasions, yielding no significant adverse events. A significant decrease in tumor size was achieved following the administration of treatment.

Zemer et al. reported results of the utilization of transcatheter arterial chemoembolization (TACE) which was employed in two cases of vascular tumors that exhibited a partial response to chemotherapy and were characterized by either a high surgical risk or recurrence after surgical resection [16]. The two patients had two huge chest tumors and underwent TACE with loading doxorubicin onto 100–300 µ m inertic spheres every 3 months as well as weekly systemic chemotherapy with vinblastine (VBL) + methotrexate (MTX). In one patient the tumor’s largest diameter reduced from 17.1 to 9 cm, and the brachial plexus was relieved of compression along with neurological and functional limb improvements. In the second patient, the tumor’s longitudinal dimension only slightly diminished, but its transverse diameter decreased by more than 50%.

Recent case series have documented favorable outcomes in the management of inoperable extra-abdominal desmoid tumors through the utilization of drug-eluting bead transarterial chemoembolization, which is loaded with doxorubicin. Elnekave et al. first successfully treated Four young patients with unresectable extra-abdominal desmoid tumors employing drug-eluting bead transarterial chemoembolization with doxorubicin during several therapy sessions [17]. Doxorubicin was chosen as the preferred chemotherapy drug based on the documented partial or complete response in individuals receiving systemic doxorubicin [22, 23]. Due to its ionic makeup, doxorubicin can be loaded into drug-eluting beads with ease. Given the recognized dose-dependent cardiotoxicity, systemic administration of doxorubicin is limited. Overall, a reduction of tumor size by 54–97%, with a size reduction of at least 30% occurring after the first treatment session in every case. Following treatment, all patients reported symptomatic improvement. On follow-up magnetic resonance imaging, T2 signal, a tumor cellularity marker, had been eliminated in three of the four patients. Cardiotoxicity or significant adverse effects were not detected.

Kim et al. also reported success with a single session of drug-eluting bead transarterial chemoembolization using doxorubicin on eleven patients with extra-abdominal desmoid tumors [18]. All of the surgeries performed were technically successful. At one month, they observed partial to almost full tumor necrosis and an overall tumor size reduction of 38.1 ± 15.3%. When compared to baseline tests, the T2 signal on follow-up magnetic resonance imaging was significantly lower after one month (4.8 ± 23.6%) and overall (29.6 ± 32%). At a six-month follow-up, ten out of the eleven (90.9%) patients reported improved symptom scores. No significant adverse effects were noticed. It is noteworthy that both investigations described brief alterations in the skin overlying tumors, which were observed in all patients and were attributed to secondary doxorubicin pigmentation.

Recently, Elnekave et al. reported the efficacy and safety of microbead-loaded doxorubicin eluting embolization (DEB-TACE) for intra- and extra-abdominal desmoid fibromatosis (DF) in 24 patients with 52 DEB-TACE sessions across six tertiary medical facilities [19]. The majority of patients (87.5%) who underwent DEB-TACE experienced partial response or stable disease, as well as a significant decrease by an average of 59% and a loss of T2 MRI intensity by an average of 36%. The surgery went well and was safe. In comparison to patients in the shorter follow-up group, patients in the longer follow-up group, who also underwent more DEB-TACE operations, had superior clinical outcomes.

**Table 1:** Summary of studies assessing the efficacy of transarterial chemoembolization (TACE) utilization in desmoid tumor

Study	Year	Study design	TACE Sample	Chemo- therapeutic agent	Total dose (mg)	Embolic material	Size reduction percentage (%)	Follow up MRI	Follow-up
Hori et al. [15]	2013	Case-report	1	Epirubicin, cisplatin	20 mg, 50 mg	50–100 μm microsphere	NA	NA	12 m
Zemer et al. [16]	2017	Case-series	2	Doxorubicin	NA	100–300 µ m inertic sphere	> 50%	NA	12–39.6 m
Elnekave et al. [17]	2018	Case-series	4	Doxorubicin	Mdn = 75 mg (R, 40 mg-125 mg)	75–150 μm microsphere	54–97%	T2 signal, a tumor cellularity marker, had been eliminated in 3 of the 4 patients.	6–32 m
Kim et al. [18]	2022	Case-series	11	Doxorubicin	Mn = 13.3 mg/m^2^ Mdn = 29.21 mg (R, 9.9 mg-76.8 mg)	100–300 μm microsphere	21–64.7%	T2 signal intensity reduction within residual tumor on the latest follow-up average of 29.6%,	3–9 m
Elnekave et al. [19]	2022	Prospective–retrospective	24	Doxorubicin	Mdn = 75 mg (R, 8 mg–269 mg)	75–150 μm microsphere	40–71%	loss of T2 intensity by an average of 36%	3–13 m
Páez-Carpio et al. [20]	2023	Case-report	1	Doxorubicin	40 mg	100–300 μm microsphere	NA	90% necrosis, evident	6 m

## Discussion

Chemoembolization is a minimally invasive therapy that targets and treats desmoid tumors by combining the principles of chemotherapy and embolization. The procedure entails the direct delivery of chemotherapeutic agents into the tumor vasculature, combined with the selective occlusion of tumor-feeding blood vessels, resulting in ischemia and cytotoxic effects on the tumor [12].

Chemoembolization offers several potential benefits in the management of desmoid tumors. Firstly, the localized delivery of chemotherapeutic agents directly to the tumor allows for higher drug concentrations at the tumor site while minimizing systemic toxicity. Additionally, chemoembolization provides a targeted approach by occluding the tumor-feeding blood vessels, resulting in a reduction in tumor size and symptomatic relief [12, 21].

By harnessing the principles of localized drug delivery and ischemic tumor damage, chemoembolization offers a promising alternative in the management of desmoid tumors. In certain patients, to be discussed later, the utilization of chemoembolization might be a safe and effective therapeutic alternative measure [18].

### Patient selection

Chemoembolization has emerged as an alternative treatment modality for desmoid tumors when conventional methods are inadequate or ineffective. To maximize the benefits and outcomes of this procedure, it is necessary to select patients with extreme care and precision. Several considerations must be taken into account during the selection procedure.

The size and location of the tumor are important factors to consider. Chemoembolization is most beneficial for localized desmoid tumors with a well-defined vascular supply. In the other hand, Chemoembolization has a limited impact on tumors with a diffuse or multifocal in character [18, 24]. Moreover, recurrent tumors are especially amenable to chemoembolization because the localized therapeutic efficacy can aid in controlling tumor growth and preventing further recurrence [17].

The patient’s clinical presentation is also crucial in determining their suitability for chemoembolization [7]. According to the Desmoid Tumor Working Group, the first-line treatment for desmoid tumors is active surveillance, and intervention should only be considered if the tumor is symptomatic or demonstrates progression [5]. Chemoembolization may be considered if the tumor responds poorly to other treatment modalities or if the morbidity associated with alternative approaches is substantial. For instance, if the tumor involves significant neurovascular pathways, surgery may be contraindicated, and chemoembolization could be a viable alternative [4].

Before contemplating chemoembolization, the overall health of the patient must also be thoroughly evaluated. Renal function must be assessed meticulously prior to procedure due to contrast-induced nephropathy. Although extremely rare, the utilized chemotherapeutic agents, such as doxorubicin, can demonstrate dose-dependent cardiotoxicity and bone marrow toxicity, despite the minimal systemic toxicity of this procedure. Therefore, patient selection should ensure that the individual has an acceptable functional status and sufficient organ function to withstand the procedure and its potential adverse effects [4, 17].

Vitally, the selection of patients for chemoembolization should be founded on an individualized evaluation performed in collaboration with a multidisciplinary team consisting of interventional radiologists, oncologists, and orthopedic surgeons. This team-based approach permits a thorough evaluation of the patient’s unique characteristics, preferences, and the treatment center’s expertise [18].

### Safety profile and complications

With all the significant benefits of chemoembolization, it is not without risks. The adverse effects can range from mild to severe, necessitating careful patient selection, risk assessment, and post-procedure monitoring. The adverse effects of chemoembolization can vary depending on several factors, including the specific technique and agent used, patient characteristics, and tumor location. Post-embolization syndrome is a common complication of chemoembolization in up to 90% of patients [29]. It presents as fever, pain, and nausea following the procedure and is generally self-limiting, lasting a few days to a week [30]. Treatment typically consists of supportive measures, such as analgesics, antiemetics, and hydration [29].

Renal complications, including contrast-induced nephropathy, can occur due to contrast agents during chemoembolization procedures [31]. Additionally, the embolization of the tumor can lead to the release of inflammatory mediators and potentially harmful substances, which can further impact renal function. Although no renal complications have yet been reported from chemoembolization of desmoid tumors, unlike in hepatocellular carcinoma (HCC), the incidence of acute kidney injury (AKI) was reported to range from 9 to 12% [32–34]. This could be attributed to the older age of onset of the disease and comorbidities in such group. Vessel injury, including arterial dissection and pseudoaneurysm formation, can occur during any endovascular procedure, including chemoembolization [35]. The occurrence of access site hematoma is an encountered complication related to the puncture site during such procedures. It is estimated to affect approximately 2% of patients [36]. In a study of more than 600 patients who underwent chemoembolization (TACE) for primary and secondary liver cancers, the incidence of vessel dissection was less than 0.5% [37]. Management of vessel injury depends on the severity and location of the injury and may require endovascular or surgical intervention [35]. Post-procedural laboratory abnormalities reported included elevated white blood count, lactate dehydrogenase (LDH), and elevated creatine phosphokinase (CPK); However, there was no evidence of renal compromise or occurrence of myoglobinuria [19].

Non-target embolization can occur due to the inadvertent migration of embolic particles or the reflux of embolic material beyond the intended target area leading to ischemia and potential tissue damage [38]. The risk is influenced by various factors, including the size and distribution of the tumor’s blood supply and the complexity of the vascular anatomy. This can result in complications such as bowel ischemia, reported in up to 2.4% of intra-abdominal chemoembolization procedures [39]. Treatment depends on the severity of the ischemia and may require surgical intervention in severe cases [38]. Preventing non-target embolization during chemoembolization is crucial to ensure patient safety and optimize treatment outcomes. Frequent identification of common arterial anatomical variants can be accomplished through CT imaging, enabling interventional radiologists to plan the arterial approach based on these imaging findings appropriately. Furthermore, the chemoembolization material is administered slowly during the procedure to prevent significant reflux and the potential occurrence of non-target embolization [40]. In addition, the use of intra-procedural Cone-Beam CT allows for angiographic mapping of the treatment area prior to chemoembolization allowing a more successful arterial selection.

The most frequently reported adverse effect following the procedure was localized pain, which occurred in more than half of the patients; this pain typically lasted for a few days and could be managed with oral analgesic medications, but some needed admission for intravenous medication to control pain and nausea [17, 18]. Chemoembolization was also associated with various dermatological manifestations, most commonly skin discolorations, which are usually self-limited with only topical treatment needed [17–19]; transient regional alopecia was also noted near the treated area and affecting wound healing and disrupting wounds which were previously healed [19]. Hence, paying particular attention to the presence of cutaneous branches susceptible to non-target embolization is crucial. Prophylactic ice topically is a commonly employed method to prevent skin complications in susceptible patients. However, it has been noted that specific individuals may experience discomfort and may necessitate the administration of additional intravenous analgesia [41]. A recent study demonstrated that inducing vasoconstriction of the prominent cutaneous arteries through subcutaneous administration of epinephrine before initiating chemoembolization can offer protection against dermatological complications [20].

### Future trends

The selective occlusion of tumor-feeding arteries by the injection of chemotherapy agents (doxorubicin or cisplatin) combined with lipiodol is the mechanism of action for conventional TACE (cTACE) [42]. By cytotoxic and ischemic actions, this causes ischemic necrosis of the target tumors. Several drawbacks from using cTACE were observed which correlated with suboptimal treatment responses in numerous cases such as [1] the fluidic mobility of lipiodol, which diminishes the effective concentrations of chemotherapeutic agents; [2] the absence of controlled and sustained drug release; and [3] heterogeneity in the approach and treatment regimens. In order to reduce these limitations, drug-eluting beads (DEBs) have been implemented as pharmacological carriers for transarterial chemoembolization (TACE). Following the administration of the beads to the intended neoplastic masses, the beads gradually and continuously release chemotherapeutic agents, such as doxorubicin or epirubicin, over an extended duration [43, 44].

DEB-TACE is a promising embolization alternative for unresectable tumors, enabling the selective occlusion of tumor-feeding arteries and subsequent interruption of blood supply to the neoplastic tissue [45, 46]. The DEB-TACE technique has the potential to gradually release and enhance the localized concentration of the antitumor agent, thereby extending the duration of retention time and improving the effectiveness of tumor necrosis [47–49]. Chemoembolization methods have evolved to increase the procedure’s effectiveness and safety. Currently, DEB-TACE is widely used to treat vital organ carcinomas that are incurable (e.g., liver [50, 51], lung [52], uterus [53], or kidney [54]) instead of cavity-based organs like the bladder and digestive system [55, 56].

The utilization of Doxorubicin-loaded DEB-TACE has been employed in the treatment of unresectable hepatocellular carcinoma, resulting in a notable increase in objective response rate (ORR) or disease control rate (DCR) [46, 50]. Furthermore, the use of superabsorbent polymer microspheres in Transarterial Chemoembolization (TACE) has demonstrated efficacy in reducing tumor size in cases of refractory lung cancer [57–59]. In a recent study, the use of doxorubicin-loaded drug-eluting beads transarterial chemoembolization (DEB-TACE) in unresectable renal cell carcinoma demonstrated a good disease control rate in the context of a short follow-up [54]. In regard to desmoid tumors, recent studies demonstrated the promising outcomes observed in transarterial chemoembolization for extra-abdominal tumors, coupled with the high level of accuracy associated with transcatheter embolization, suggest that employing transarterial chemoembolization as a treatment modality for intra-abdominal tumors could potentially eliminate the necessity for a potentially hazardous surgical resection [13]. Despite the long-standing development of drug-eluting microspheres, the incorporation mechanism poses limitations on the selection of drugs that can be utilized [60]. In general, microspheres employ either an ion-exchange technique or a swelling procedure, subsequently facilitating the interaction between the drug and ionized side chains (e.g., carboxylate or sulfonate groups) in order to accomplish drug loading [61, 62]. Hence, it is plausible to incorporate solely drugs with positive charges and low molecular weights, such as DOX, Idarubicin, and Irinotecan [63]. Additionally, the sizes of a variety of currently available microspheres range from 30 to 900 m [63]. Still unable to penetrate arterial capillaries, they decrease medication penetration and the overall tumor volume exposed to treatment [60]. With the exception of enhancing patient acceptability and lowering systemic drug responses, DEB-TACE therapy does not appear to offer any further benefits in terms of efficacy than c-TACE therapy, according to an abundance of research [64–66]. While the aforementioned therapy has exhibited promising outcomes with a good level of safety. Further research is required to assess the most effective therapeutic approach for patients with desmoid tumors through the implementation of additional prospective, randomized, controlled trials with sufficient statistical power.

The use of nanomaterials in treatment has received a lot of interest recently [67–69]. Nanomedicine has presented innovative opportunities for addressing current challenges in transarterial chemoembolization (TACE) therapy. The unique attributes of nanomaterials, including nanoscale carriers, a notable specific surface area, and distinct physicochemical properties, render them highly suitable for drug delivery goals [70], improvement of pharmaceutical properties [71], precise targeting [72], The concurrent administration of multiple drugs [73], and the integration of imaging modalities with drug delivery techniques enables the visualization and localization of drug delivery sites [74, 75]. These studies have unquestionably provided a larger vision for clinical TACE therapy of cancer, even though the multifunctional nanoplatforms have not yet been widely used in clinical practice, and several challenges that restrict the efficacy of TACE also need to be solved promptly [76]. Additionally, the possibility of several novel nanomedicine-based cancer therapies, like molecular dynamic therapy (MDT) [77], chemodynamic therapy (CDT) [78], provides researchers with novel possibilities and difficulties to create some inventive multifunctional nanoplatforms that will increase the effectiveness of TACE in the treatment of cancer. These nanoplatforms’ design principles, functional attributes, and therapeutic properties served as a foundation for the creation of more suitable and efficient embolization materials. In light of these previous studies, it is therefore strongly anticipated that new multifunctional nanoplatforms with improved efficacy and safety will be developed and utilized in TACE therapy for cancer [76].

### Study limitations

This systematic review’s limitations underscore the necessity for more research to confirm the efficacy of transarterial chemoembolization (TACE) in the management of desmoid tumors. Significantly, there were no randomized controlled trials (RCTs) available, and the majority of the included research were case reports or case series, which inherently possess a greater risk of bias and restrict the capacity to derive conclusive findings. The limited sample sizes in the examined studies further constrain the generalizability of the results. The absence of control groups for comparison, including individuals undergoing standard treatments or no treatment, further constrains the capacity to ascertain the relative advantages and disadvantages of TACE. Furthermore, the lack of long-term data hinders a comprehensive assessment of the treatment’s effectiveness, recurrence rates, and safety profile.

## Conclusion

In summary, this narrative review demonstrates the prospects of chemoembolization as a beneficial alternative for managing desmoid tumors, despite the scarcity of relevant studies. It presents itself as a promising alternative, demonstrating notable effectiveness and limited adverse effects, rendering it a feasible selection, especially in cases when traditional therapies are inadequate or ineffective. The primary factors to be considered for its utilization encompass the dimensions and placement of the tumor. Tumor size and location are key considerations. Chemoembolization is better for large tumors or tumors that are in anatomically difficult areas for surgical access, as surgical removal may pose higher risks or result in incomplete removal. In addition, malignancies that exhibit well-defined vascular supply are considered optimal candidates for this method, since it allows for targeted delivery of chemotherapy and mitigates the risk of systemic toxicity. This review underscores the importance of chemoembolization as a beneficial treatment option for desmoid tumors, offering renewed hope for patients dealing with these intricate cases.

## Data Availability

No datasets were generated or analysed during the current study.

## Appendices

### Appendix A

**Table:**

Database	Search syntax/ string used
PubMed	((((((((Transcatheter arterial chemoembolization[Title/Abstract]) OR (TACE[Title/Abstract])) OR (transarterial chemoembolization[Title/Abstract])) OR (chemoembolization[Title/Abstract])) OR (drug eluting beads[Title/Abstract])) OR (DEB-TACE[Title/Abstract])) OR (embolization[Title/Abstract])) OR (emboli*[Title/Abstract])) AND ((((((((((((((desmoid[MeSH Terms])) OR (Deep fibromatosis[Title/Abstract])) OR (Desmoid fibromatosis[Title/Abstract])) OR (Desmoid[Title/Abstract])) OR (desmoid tumor[Title/Abstract])) OR (desmoid-type fibromatosis [Title/Abstract])) OR (aggressive fibromatosis [Title/Abstract])) OR (fibromatosis[Title/Abstract])) OR (Musculoaponeurotic fibromatosis [Title/Abstract]))
EMBASE	((((((((‘Transcatheter arterial chemoembolization’:ti, ab) OR (TACE: ti, ab)) OR (‘transarterial chemoembolization’:ti, ab)) OR (chemoembolization: ti, ab)) OR (‘drug eluting beads’:ti, ab)) OR (DEB-TACE: ti, ab)) OR (embolization: ti, ab)) OR (emboli*:ti, ab)) AND ((((((((((((((desmoid/exp)) OR (‘Deep fibromatosis’:ti, ab)) OR (‘Desmoid fibromatosis’:ti, ab)) OR (Desmoid: ti, ab)) OR (‘desmoid tumor’:ti, ab)) OR (‘desmoid-type fibromatosis’:ti, ab)) OR (‘aggressive fibromatosis’:ti, ab)) OR (fibromatosis: ti, ab)) OR (‘Musculoaponeurotic fibromatosis’:ti, ab))))))
Google Scholar	Keywords used included “chemoembolization”, “TACE”, “Drug-eluting beads transarterial chemoembolization”, “doxorubicin-eluting beads transarterial chemoembolization”, “DEB-TACE” AND “Desmoid tumor”, “aggressive fibromatosis”

### Appendix B

**Table:**

JBI Checklist	Hori et al. [14]	Zemer et al. [15]	Páez-Carpio et al. [19]
Were patient’s demographic characteristics clearly described?	?	+	?
Was the patient’s history clearly described and presented as a timeline?	+	+	+
Was the current clinical condition of the patient on presentation clearly described?	+	+	+
Were diagnostic tests or assessment methods and the results clearly described?	+	+	+
Was the intervention(s) or treatment procedure(s) clearly described?	+	+	+
Was the post-intervention clinical condition clearly described?	+	+	+
Were adverse events (harms) or unanticipated events identified and described?	+	+	+
Does the case report provide takeaway lessons?	+	+	+

**Table:**

JBI Checklist	Elnekave et al. [16]	Kim et al. [17]
Were there clear criteria for inclusion in the case series?	+	+
Was the condition measured in a standard, reliable way for all participants included in the case series?	+	+
Were valid methods used for identification of the condition for all participants included in the case series?	+	+
Did the case series have consecutive inclusion of participants?	+	+
Did the case series have complete inclusion of participants?	+	+
Was there clear reporting of the demographics of the participants in the study?	+	+
Was there clear reporting of clinical information of the participants?	+	+
Were the outcomes or follow up results of cases clearly reported?	+	+
Was there clear reporting of the presenting site(s)/clinic(s) demographic information?	+	?
Was statistical analysis appropriate?	+	+

**Table:**

JBI Checklist	Elnekave et al. [18]
Were the two groups similar and recruited from the same population?	+
Were the exposures measured similarly to assign people to both exposed and unexposed groups?	-
Was the exposure measured in a valid and reliable way?	+
Were confounding factors identified?	+
Were strategies to deal with confounding factors stated?	+
Were the groups/ participants free of the outcome at the start of the study (or at the moment of exposure)	NA
Were the outcomes measured in a valid and reliable way?	+
Was the follow up time reported and sufficient to be long enough for outcomes to occur?	+
Was follow up complete, and if not, were the reasons to loss to follow up described and explored?	+
Were strategies to address incomplete follow up utilized?	+
Was appropriate statistical analysis used?	+

+ yes, - no, ? unclear, *NA* not applicable.
